# Preliminary Evaluation of the Step‐by‐Step Parenting Program for Expfectant Parents With Intellectual Disabilities

**DOI:** 10.1111/jar.70034

**Published:** 2025-03-20

**Authors:** Maurice A. Feldman, Amanda Cappon, Kay Corbier, Vicky Caruana, Mechane Laronde, Kendra Thomson

**Affiliations:** ^1^ Dept. of Applied Disability Studies Brock University St. Catharines Canada; ^2^ Faculty of Social and Community Services Durham College Oshawa Canada; ^3^ Developmental Services Worker Program Durham college Oshawa Canada; ^4^ Starfish Parent Support Program Courtice Canada

**Keywords:** expectant parents, intellectual disabilities, parenting program

## Abstract

**Background:**

This study evaluated the Step‐by‐Step Parenting Program (SBSPP) to prepare expectant parents with intellectual disabilities to care for their newborns.

**Method:**

Two expectant parents with intellectual disabilities were seen once or twice weekly in their homes for about 2 h over 16 and 20 weeks (21 and 27 sessions), respectively. The key measure was percentage correct scores on parenting skill checklists with an infant simulator (IS) and eventually the newborn. Newborn‐care skills trained were sponge bath, dressing, ear and nose care, and treating cradle cap. SBSPP‐IS training consisted of instructions, prompting, modelling, and feedback. Multiple baselines across skills and participant designs were used.

**Results:**

Trained skills increased and transferred to the newborns. Both parents have maintained custody of their infants for over 1 year.

**Conclusion:**

This study provides preliminary evidence that prenatal parent training may help expectant parents with intellectual disabilities to properly care for their newborns.

**Summary**

- Expectant parents with intellectual disabilities may want to practice taking care of a baby before they give birth.
- In this study, two mothers‐to‐be received training on some skills needed to take care of a newborn like giving a sponge bath and dressing, using a life‐like infant simulator instead of a real baby.
- The parents learned the skills and when their babies were born, the parents were able to immediately use the skills they learned with their babies without needing more training.
- This study showed that expectant parents with intellectual disabilities can learn parenting skills using an infant simulator before their baby is born and transfer what they learned when taking care of their baby.

## Introduction

Child neglect is one of the most serious public health concerns yet often receives far less attention than physical and sexual abuse (National Scientific Council on the Developing Child 2012). Child neglect is related to poverty and has lifelong physical and mental health implications for the child (National Scientific Council on the Developing Child 2012; Rozanski et al. 2021). In the U.S., child neglect makes up 76% of all child protection cases and 78% of child maltreatment fatalities (U.S. Department of Health, and Human Services, Administration on Children, Youth, and Families, Children's Bureau 2023). There are numerous legal and scientific definitions and different types of neglect, but a common element is the failure of the parent to meet the child's needs related to basic care, safety, health, and emotional, social, language, and cognitive development. Early intervention may overcome negative sequelae due to neglectful care (e.g., insecure attachment, paediatric health problems, developmental delay) and lead to positive parent–child relationships and family reunification (Feldman et al. 1992a, 1993; National Scientific Council on the Developing Child 2012).

One group of parents who are considered at risk for child neglect is parents with intellectual disabilities. Although reproductive rights of persons with disabilities are enshrined in Article 23 of the UN Convention on the Rights of Persons with Disabilities (2006), it is still the case that these parents are disproportionally represented in child protection cases and have their parenting rights terminated, often without evidence of child maltreatment or provision of appropriate services (DeZelar and Lightfoot 2018; McConnell et al. 2011; Kendrick 2024; LaLiberte et al. 2017; Rebbe et al. 2021). It is now recognised by experts in the field that IQ is not a strong predictor of parenting competence and a more nuanced contextual model, such as the one elucidated by Feldman (2002), provides a more accurate prediction than parental IQ of which parents will and will not be able to successfully care for their children (IASSID Special Interest Research Group on Parents and Parenting with Intellectual Disabilities 2008; McGaw et al. 2010). Contextual variables such as the parent's history (e.g., trauma, stigmatisation); poor health; economic hardship; social isolation; lack of appropriate supports, parenting education, child‐care experiences and parenting role models; and societal and worker negative biases towards parenting by persons with intellectual disabilities (among other factors) impact parent, child and family outcomes (Emerson et al. 2015; Feldman and Aunos 2020; Feldman et al. 2012; Granqvist et al. 2014; Hammarlund et al. 2023; Hindmarsh et al. 2017; McConnell et al. 2011, 2021, 2022; Wade et al. 2011, 2015).

Recent research has focused on how these contextual variables affect maternal health, birth, and newborn outcomes when mothers have intellectual disabilities. Large national, regional, and targeted surveys from several countries (Canada, Sweden, U.K., U.S.) have shown that compared to expectant mothers without intellectual disabilities, mothers‐to‐be with intellectual disabilities were more likely to be younger; smoke; eschew, delay or not benefit from prenatal care resources; and had more pregnancy and birth complications, such as induced labor, caesarean section and death (Brown et al. 2016; Homeyard et al. 2016; Mitra et al. 2019). Their newborns were more likely to be stillborn, premature/low birth weight and have low Apgar scores (Feldman and Aunos 2020; Höglund and Larsson 2019; Mitra et al. 2015). Increased risk of mother and child physical, developmental and psychological problems persisted postnatally, such as maternal postpartum psychiatric hospitalizations and early childhood injuries and developmental delay (Brown et al. 2017; Feldman et al. 1985; Wickström et al. 2017). These findings suggest that the prenatal and postnatal periods are crucial to promoting subsequent infant health and development and may contribute to reasons why children of parents with intellectual disabilities are at risk for early and subsequent health, developmental and behaviour problems (Feldman et al. 1985; Feldman and Walton‐Allen 1997; McConnell et al. 2003).

Prenatal services for pregnant parents with intellectual disabilities may alleviate some of the concerns raised by recent studies of perinatal outcomes in parents with intellectual disabilities and their children. Prenatal applications of the U.S.‐based Early Head Start for low‐income and other at‐risk families (but not specifically parents with intellectual disabilities) contributed to some positive child‐care and developmental outcomes above postnatal‐only versions (Keilty and Smith 2020; Love et al. 2002). Unfortunately, little attention has been paid to the prenatal experiences of and services for expectant parents with intellectual disabilities (Homeyard et al. 2016). Potvin et al. (2020) reported two case studies that illustrated the lack of availability of accessible perinatal information for parents with intellectual disabilities. Two qualitative studies of pregnant women with intellectual disabilities described the importance of informal and formal support systems in promoting various aspects of self‐advocacy, pregnancy, childbirth and preparation for becoming a parent (Potvin et al. 2016; Rosenthal et al. 2022). While we could find no quantitative evaluations of prenatal parenting skill training programmes for parents with intellectual disabilities, Höglund and Larsson (2019) ran a randomised control trial (RCT) with Swedish high school students with intellectual disabilities in which students attended a 13‐week family life class that included a Parenting Toolkit (ASVS 2022) designed to expose the students to the realities and practicalities of taking care of children. They also cared for a;3‐month‐old’ infant simulator for 3 days and nights that was programmed as ‘hard’ (to care for). Quality of care measures were automatically recorded by the simulator, but these data were not presented. Overall, the results showed that the students who attended the course reported more knowledge of and had more realistic attitudes about parenting; they were more likely to make informed decisions about parenthood compared to the control group.

From our review of the literature, it appears that no study has yet evaluated teaching specific newborn and infant care skills to expectant parents with intellectual disabilities using an infant simulator and then testing the impact of prenatal training when the baby was born. Prenatal parent education interventions could capitalise on the wealth of evidence of effective parent training for parents with intellectual disabilities. Since the first review of interventions 30 years ago (Feldman 1994) and confirmed in subsequent reviews (Wade et al. 2008), we know that key elements of effective parent education for parents with intellectual disabilities include (1) task analysis of child‐care skills and parent–child interactions; (2) objective observations and teaching of parenting skills in natural settings; (3) behavioural skills training (instructions, modelling, prompting, roleplaying, performance feedback) and (4) programming for generalisation and maintenance (Feldman and Tahir 2016). The Step‐by‐Step Parenting Program SBSPP, (Feldman and the Surrey Place Parenting Enhancement Program 2024) started in the 1980's and is probably the most researched parenting education program for parents with intellectual disabilities, with over 30 peer‐reviewed articles and book chapters. The SBSPP evaluations, using single‐case experimental designs for example, (Feldman et al. 1992, 1997) and RCTs (Feldman et al. 1992, 1993) showed quick improvements in the parents' basic child‐care skills, child health and safety skills and positive parent–child interactions to training criterion levels comparable to parents who were without disabilities and had no child protection involvement (Feldman 1998). When the parents increased their skills, corresponding benefits accrued to the children's health and development and far fewer parents lost custody of their children (e.g., Feldman et al. 1992, 1992, 1993, 1997). While workers anecdotally have reported that SBSPP has been used successfully with expectant parents with intellectual disabilities, there are yet no peer‐reviewed experimental evaluations of the impact of the SBSPP with these parents‐to‐be using an infant simulator.

The research questions were (1) could the Step‐by‐Step Parenting Program coupled with an infant simulator increase parenting skills in expectant parents with intellectual disabilities; (2) would training effects transfer to the new baby; (3) would there be an increase in parenting self‐efficacy after training and (4) would the participants be satisfied with the training?

## 1 Methods

### 1.1 Ethics Clearance

This study received ethics clearance from the Research Ethics Boards of Durham College and Brock University. The Research Ethics Boards conform to the most recent Canadian Tri‐Council Policy Statement: Ethical Conduct for Research Involving Humans—TCPS 2 (2022)—https://ethics.gc.ca/eng/policy‐politique_tcps2‐eptc2_2022.html.

### 1.2 Conflict‐of‐Interest Statement

The first author receives compensation when he conducts training for workers, but he received no compensation for the training conducted for this study. The fourth and fifth authors received compensation as the parent educators in this study. The other authors report no conflict of interest.

### 1.3 Participants

Note that the participants' names are pseudonyms, and we are using the parents' requested gender and parent identity designations. Four expectant parents with intellectual disabilities (three mothers) or autism (one father) were recruited and consented to participate. One mother dropped out due to health issues, and the father decided not to receive training. The remaining two participants (Wendy and Sara) were in female–male cohabiting relationships, unemployed and receiving government financial assistance for persons with disabilities. We did not ask their exact ages, but they indicated that they were between 20 and 35 years old. Wendy identified as female of Southeast Asian descent and was born in Canada. She has intellectual disabilities and reported a history of emotional abuse and being bullied. Sara was born in Canada and identified as a Caucasian female. She has intellectual disabilities and Fragile X syndrome. She reported a trauma history of physical and emotional abuse, neglect, witnessing family violence and being bullied.

### 1.4 Pregnancy and Birth Complications

Both families were expecting their first child. At the start of the first baseline probe, Wendy and Sara were 33 and 36 weeks pregnant, respectively. Wendy had gestational diabetes in the second trimester that required blood monitoring but no medication. She gave birth early and naturally at 37.6 weeks gestation; Sara had no issues during her pregnancy until the fetus heart rate spiked, prompting an emergency caesarean section 8 days after her due date.

### 1.5 Consent

Both participants received a copy of the consent form written in simple language. A research assistant read the consent form out loud, asked the participants questions to test comprehension and offered them the opportunity to ask questions. Both participants understood and signed the consent form.

### 1.6 Home Visits

The study took place in each family's home, through once or twice‐weekly visits that lasted approximately 2 h. From the first baseline to the last follow‐up session, Wendy had a total of 21 sessions over 16 weeks and Sara had 27 sessions over 20 weeks. Some sessions were cancelled at the request of the parent or due to the unavailability of the parent educator or research assistant, the birth of the baby and the Christmas holiday period.

### 1.7 Parent Educators

Two parent educators conducted observational probes and training. The parent educator who worked with Wendy had a Social Service Worker Diploma, 25 years' experience in intellectual disabilities and 3 years of experience with parents who have intellectual disabilities. The second parent educator, who trained Sara, had an Early Childhood Education Diploma, 35 years' experience working with persons who have intellectual and developmental disabilities, and 16 years of experience with parents who have intellectual disabilities. Prior to the start of the study, both parent educators attended an in‐person three‐day training on the Step‐by‐Step Parenting Program run by the first author. They then received follow‐up virtual meetings and emails to be trained on the research protocols, including how to score the checklists and conduct the skills assessments, probes and training. The first author corresponded frequently with the parent educators (and research assistants) to maintain research integrity and answer any questions that arose.

### 1.8 Experimental Design

Combined multiple baselines across skills and participants designs were used to demonstrate a cause‐and‐effect relationship between the parent training intervention and changes in trained parenting skills (Cooper et al. 2020). Cause‐and‐effect control is established through time‐series data collection by staggering the initiation of training across skills within and across participants. The replicated finding that skills only increase and maintain at high levels when training is provided demonstrates experimental control of the independent variable (training) over the dependent variable (measure of parenting skills). The advantages of this design include not having to run many participants to demonstrate cause‐and‐effect relationships between the independent and dependent variables, nor requiring a no‐treatment control group (as in a between‐groups design) or withdrawing treatment (as in a reversal design). A disadvantage of small‐*N* studies is that numerous single‐case experimental design replications are needed to demonstrate the generalisability of findings. Single‐case experimental designs have been used frequently in research evaluating parenting interventions for parents with intellectual disabilities see reviews by (Feldman 1994; Wade et al. 2008).

### 1.9 Infant Simulator

The infant simulator used in this study is the Real Care Baby https://www.realityworks.com/product/realcare‐baby‐3‐infant‐simulator/?v=c86ee0d9d7ed. It weighs 3.18 kg (7lbs) and is 53.34 cm (21 in.) in height. The researchers' original intention was to program the Real Care Baby on easy mode (some squirming, possible crying), but when shown to the participants, they both requested that the training be conducted with the simulator turned off.

### 1.10 Assessment and Selection of Skills Trained

Both participants received training on giving a newborn a sponge bath. Wendy was also trained on infant ear and nose care. Sara was also trained on dressing an infant and treating cradle cap in a newborn. These skills were chosen through a collaborative process. The parent educator reviewed with the participant up to 104 skills in the SBSPP curriculum appropriate for newborns and infants, and the participant then selected at least five skills on which she wanted training. The participant's performance on these skills was assessed during the pre‐training stage using the SBSPP Checklists (see below). The parent educator provided any needed supplies (e.g., diaper, sponge) and then asked the parent to perform the skill with the infant simulator using probe procedures described below under parenting skills measurement. Skills with high checklist scores (≥ 80%) were not considered in need of training, based on scores of parents without disabilities and child protection involvement (Feldman 1998). More skills were added to the skills assessment to replace the skills not needing training. Each participant chose three skills that they wanted training on first from the remaining skills with low (< 60%) pretraining probe scores. Wendy originally had three identified skills for training, but infant fingernail care using a nail clipper was dropped at her request because she said she was uncomfortable using the nail clipper on the infant simulator and would not use it when their baby was born.

### 1.11 Child‐Care Skill Checklists

SBSPP child‐care skill checklists applicable for newborns and infants were used to measure the parents' performance, first with the infant simulator and then with the baby. Most of the SBSPP checklists are task analyses of common child‐care skills applicable from birth to about 36 months of age (with additional checklists covering older children). The checklists were vetted by paediatric health care professionals and are kept up to date regarding child‐care research and best practice. All published SBSPP studies using single‐case experimental designs have shown the checklists to have acceptable test–retest reliability (i.e., baseline scores across observations remain stable); interobserver agreement (between trained, independent observers); distinguish parents who need training on specific skills from those who do not; and are sensitive to training effects (e.g., Feldman 1998; Feldman et al. 1985, 1993, 1992a, 1992b). An example of the Treating Cradle Cap checklist is provided in Table 1.

**TABLE 1:** Example of a step‐by‐step parenting program parenting skills checklist.

	STEP‐by‐step treating cradle cap checklist	**✓**/X/NA/NO	Comments
1.	Before shampooing, applies small amount of petroleum jelly or mineral/baby/olive oil to baby's scalp and lets absorb for a few minutes to several hours		
2.	Uses a soft brush or toothbrush to gently remove scales from the baby's scalp		
3.	Places small amount of baby shampoo or soap onto their hands		
4.	Mild steroid cream or antifungal shampoo is used only with doctor's approval (if applicable)		
5.	Gently rubs baby's head with hands		
6.	Rinses baby's head with warm water from cup, wet cloth, or cupped hand		
7.	While rinsing, places hand across baby's forehead or tilts head back		
8.	Dries by gently patting head with clean towel		
9.	Gently rubs head with a few drops of oil		
10.	Combs hair gently (if child is older than 6 months)		
	Condition of Scalp:		
	Source of Information:		
	Total no. of **✓**		
	Total no. of steps **✓ + X**		
	Percentage correct = (total no. **✓**/total steps **✓ + X**) times100%)		

### 1.12 Measures

#### 1.12.1 Parenting Skills

The primary dependent measure was percentage correct on trained skills through direct observation (probes) in the home using SPSPP skills checklists. Each step of the checklist was scored correct (**✓**); incorrect (**x**)—either performed incorrectly or not at all; or not applicable (**NA**)—because the parent did not have the opportunity to perform the skill. For instance, if a physician did not prescribe or recommend a steroid cream or antifungal shampoo, then step 4 of the Treating Cradle Cap checklist (Table 1) would be scored NA. In addition, when observations were made from videos, we added a **NO** (not observable) score. For example, the first step on the sponge bath checklist says, ‘room is warm and draft free’. As this observation could not be determined from the video, this step would be scored NO. An overall checklist percentage was calculated as no. correct/no. correct + no. incorrect converted to a percentage. NA's and NO's were not included in the percentage correct score. Observational probes occurred prior to any training on each visit, across all conditions (baseline, training and follow‐up) and were videorecorded by a research assistant. Wendy's probe videos were scored by a research assistant who was aware of the phase of the study being scored. Sara's parent educator scored the probes in the moment. No training was supposed to occur during the probes (see Probe Integrity, below, for minor exceptions). To conduct a probe, we asked the parent to perform the skill being measured and they were thanked afterwards for letting us observe them.

#### 1.12.2 Interobserver Agreement (IOA)

The first author trained two research assistants to conduct IOA checks. The IOA checkers then practiced on videos from the skills assessments until they reached at least 85% agreement across three consecutive videos. As each research assistant accompanied one parent educator to home visits for Wendy or Sara, respectively, they conducted probe IOA on the other participant who they did not meet. The research assistants were naïve to the phases of the probe videos and the previously obtained probe scores they were scoring. Wendy's probe scores were compared to another non‐naïve research assistant's scores from the videos. For Sara, the research assistant's IOA scores were compared to the parent educator's in‐person probe scores. Probe IOA was scored on each step of the designated skill checklists across both participants and the baseline, training and follow‐up phases, including with the baby in follow‐up (baby probes). An agreement was defined when both observers scored a ✓, x, NA or NO on a step. A disagreement occurred if the two observers put different scores on a step. Item‐by‐item IOA was summarised as no. agreements/no. agreements + no. disagreements, converted to a percentage. Videos used for IOA (as well as treatment and probe integrity) checks were chosen at random using an online random number generator (https://www.calculator.net/random‐number‐generator.html). Overall, IOA was conducted on 42% of probes across participants and phases; mean IOA equalled 90% (range: 50%–100%). IOA for baseline was 91% (range: 50–100); training—89% (range: 50–100); follow‐up with infant simulator—91% (range: 87–96) and baby probes—89% (range: 83–100). IOA for sponge bath was 90% (range: 82–96); ear and nose care—89% (range: 50–100); dressing—91% (range: 86–100) and treating cradle cap—91% (range: 80–100).

#### 1.12.3 Treatment Integrity

As mentioned, the parent educators were trained on the SBSPP training protocols. Their performance in implementing the training was scored by a research assistant using the checklist found in Table 2. Each item was scored correct (**✓**), incorrect (**x**), not applicable (**NA**) or not observable (**NO**). Treatment integrity was scored on 57% of training videos chosen at random and was 100% across both parent educators. Treatment integrity IOA was conducted on 9 of the 14 (56%) randomly selected scored treatment integrity videos by an independent observer. Treatment integrity IOA was 98.78% (range: 93%–100%).

**TABLE 2:** Behavioural skills training integrity checklist.

1.	Parent educator (PE) is prepared—brings checklists, materials, and props needed to do probe observations and training.
2.	PE conducts observation probes and records results on checklists (unless probes are video recorded for future scoring).
3.	PE starts training by going over the checklist steps with the parent. PE first indicates which steps parent performed correctly and praises parent. Then PE goes over which steps there is room for improvement.
4.	PE slowly models task and emphasises correct way to perform missed or incorrect steps and explains while modelling.
5.	PE then allows parent to try again as soon as possible after the model, records performance on the checklist and provides prompts and positive feedback, as the parent performs.
6.	PE gradually intersperses non‐judgmental, but specific corrections.
7.	PE gives positive feedback statement(s) for correct responses.
8.	PE debriefs parent within 1 min of task completion going over what the parent did correctly, where the parent improved and what the parent needs to practice.
9.	PE ends training on a positive note about the parent's performance.

#### 1.12.4 Probe Integrity

The same research assistant who conducted treatment integrity checks also viewed 36% of the study probe videos chosen at random and recorded whether any training components (e.g., instructions, prompts, models, performance feedback) occurred during the probes. It was found that 6 of 45 probes (13%) had minor training components, all with Sara. Three times the parent educator had to intervene for safety reasons when the parent forgot to check the bath water temperature before giving the infant simulator a sponge bath. There were two probes when the parent educator said, ‘good job!’ once, and another probe where the parent educator offered advice. As these probes included only a brief instruction, feedback or information, and their scores were not outliers, it was decided to include them in data display and analysis. IOA on probe integrity was conducted by an independent observer who scored 14 of the 45 (31%) of the scored probe integrity videos, selected at random. This observer was blind to the phase of the video being scored. Probe integrity IOA was 100%.

#### 1.12.5 Parenting Self‐Efficacy

As seen in Table 3, we asked the participants before training and after follow‐up 11 questions adapted from the Parent Sense of Competence Scale (Gibaud‐Wallston and Wandersman 1978), cited in (Johnston and Mash 1989) and questionnaires used in another Canadian research project (McConnell et al. 2022). Each question was scored on a rating scale of 1 = ‘Not at all confident’ to 5 = ‘Very Confident’. The pre‐training questionnaires were administered in person by the same research assistants who obtained consent. The post follow‐up questionnaires were conducted virtually by two researchers (one for Wendy and one for Sara) who the parents had not previously met.

**TABLE 3:** Parent self‐efficacy questionnaire.

1.	I am confident I know what my child needs
2.	The things I do will make a difference in my child's behaviour
3.	I can make an important difference in my child's life
4.	Being a parent is manageable, and any problems are easily solved
5.	When I feel anxious or tense about being a parent, I feel that I am able to manage these emotions
6.	Being a good parent is a reward in itself.
7.	I honestly believe I have all the skills necessary to be a good parent to my child.
8.	Being able to distinguish between knowing whether I'm doing a good job or a bad one
9.	I am motivated to do a good job as a parent
10.	Even though I may not always manage, I will know when to ask for help with my child
11.	How confident are you in your abilities as a parent overall?

*Note:* On a scale of 1 (Not at all confident) to 3 (Neutral) to 5 (Very confident), please rate how confident you feel about being a parent.

#### 1.12.6 Participant Satisfaction

After follow‐up, the same researchers who administered the virtual post‐follow‐up parenting self‐efficacy questionnaires also asked the parents to rate their satisfaction with their participation in the training study. The rating scale ranged from 1 = Not at all satisfied to 5 = Extremely satisfied. The rating questions were: (1) How satisfied do you feel that the parenting program was helpful in terms of preparing you to take care of your baby? (2) Please rate each skill you learned in the Step‐by‐Step training on how satisfied you feel that the program has prepared you to care for your baby (the parent rated each of the skills on which they were trained). (3) Please rate your satisfaction with the parent educator's support through this skills training. In addition, the parents were asked three yes/no questions: (1) Did you feel comfortable asking the parent educator questions? (2) If you asked questions, did you feel those questions were answered to your satisfaction? (3) Was the parenting program geared towards your learning style (step‐by‐step, demonstrations, coaching)? Finally, the parents were asked to describe what they thought of their participation, their favourite part of the parenting program, and what they felt they learned that was most helpful to them as a new parents. Their responses were written down verbatim.

### 1.13 Procedure

#### 1.13.1 Pre‐Training

After the parent consented to participate, a research assistant collected demographic information and administered the parent self‐efficacy questionnaire. Subsequently, the assigned parent educator and research assistant met with the participant in their home to identify target training skills through parenting skills assessments using the infant simulator (data not presented). The procedure for identifying target skills is described in the Assessment and Selection of Skills Trained section.

#### 1.13.2 Baseline

Prior to the start of training, each participant received a minimum of four baseline probes on the first trained skill—giving a newborn a sponge bath.

#### 1.13.3 Step‐by‐Step Training

Each home visit during the training phase started with probes of skills remaining in baseline, being trained, or in follow‐up. For skills being trained, these probes served as maintenance checks from the previous visit. Wendy and Sara received the behavioural skills training (BST) package. BST components are described in Table 2. If home visit time allowed, the parent practised again and received BST. The parents did not want to keep the infant simulator at home to practise between visits.

#### 1.13.4 Follow‐Up

When probe performance on a skill in training reached ≥ 80% across at least two consecutive probes, then that skill entered follow‐up and training ceased on that skill. However, if in follow‐up, the skill dropped below 80% and/or the parent requested it, booster training could be offered. The booster typically was abbreviated BST and focused on incorrect steps from the previous probe. Sara requested and received two boosters for sponge bath and dressing. After the babies were born, follow‐up probes were conducted with the actual baby. Follow‐up for sponge bath and ear and nose care lasted 5 and 1 week, respectively, for Wendy. Follow‐up for sponge bath, dressing, and treating cradle cap lasted 18, 16 and 4 weeks, respectively, for Sara. After the final follow‐up visit, researchers not involved in training administered the post‐parent self‐efficacy and consumer satisfaction questionnaires in separate video meetings with Wendy and Sara, respectively.

## 2 Results

### 2.1 Parenting Skills

**FIGURE 1:**
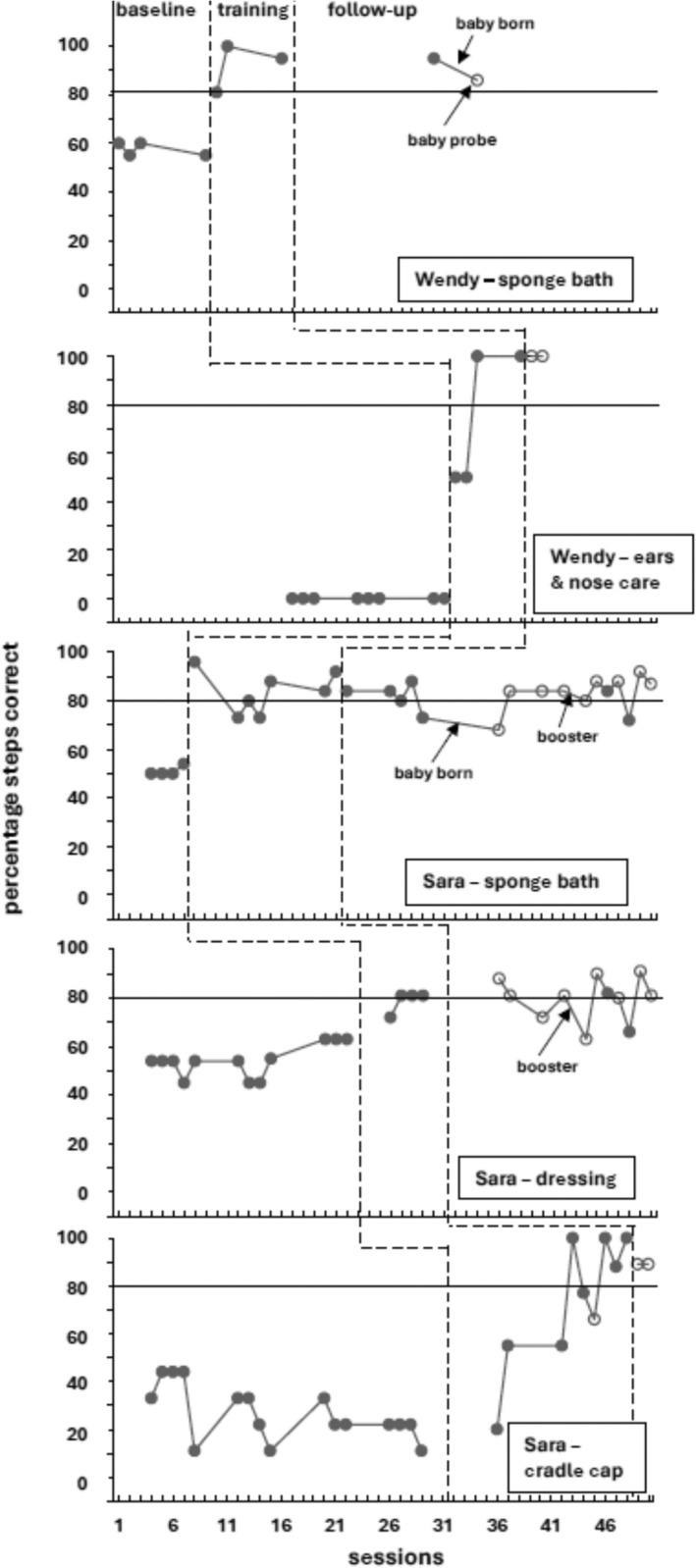
Concurrent multiple baseline across skills and participants designs probe results for both participants' percentage parenting skill steps correct across baseline, training and follow‐up. Closed circles are probes with the infant simulator and open circles are with their babies. The horizontal solid line at 80% represents the training criterion.

Figure 1 shows the concurrent multiple baseline designs results for the two participants, as well as when the babies were born. All data points in the training phase reflect probes conducted prior to a training session on the same visit. Note that Wendy started training on ear and nose care that continued after the baby was born. Sara received training on treating cradle cap after her baby was born. All postnatal training was with the infant simulator for both participants. Parenting skills increased during training with the infant simulator and, for the most part, transferred to the baby (open circle baby probes). The overall mean phase scores across participants and skills increased from a baseline of 31.04%–79.09% in training, and 82.09% in follow‐up with the infant simulators, and 84.61% in follow‐up with the baby. Table 4 shows the mean percentage scores across skills for baseline, training and follow‐up phases for both participants. Baby probes occurred after the parents reached training criterion with the infant simulator, except that Sara had one baby probe (session 42 in Figure 1) while still in training for treating cradle cap with the infant simulator. Wendy and Sara's mean baby probe scores across skills were 95.33% and 82.12%, respectively; 83% of their baby probes were ≥ 80%.

**TABLE 4:** Mean percentage scores across phases and skills for the two participants.

	Baseline	Training	Follow‐up‐simulator	Follow‐up‐baby
Wendy—sponge bath	57.5%	92%	95%	86%
Wendy—ear and nose care	0	75		100
Sara—sponge bath	51	83.71	80.71	83.89
Sara—dressing	52.3	73.5	74	80.78
Sara—cradle cap	26.81	74.38		89

Effect size estimates regarding changes in skill performance between baseline and training, and baseline and follow‐up with the infant simulator and baby probes, respectively, were calculated for each participant and skill using the Baseline Corrected Tau statistic (Tau‐BC, Tarlow 2017). Tau‐BC is used in single‐case time series data with baseline and treatment phases. Tau‐BC is a nonparametric statistic suitable for small sample sizes and may be less sensitive to serial dependency and outlying data points than other effect size measures (Dowdy et al. 2021). The calculation first removes the confound of any statistically significant baseline trends (increasing or decreasing) using Kendall rank‐order correlation that may cloud the interpretation of a treatment effect size. A rank‐order Tau correlation between baseline and treatment scores then is calculated, yielding a coefficient between −1 and +1 (Pustejovsky et al. 2024; Tarlow 2017). Vannest and Ninci (2015, 408) suggested the following benchmark interpretation for Tau statistics: 0.0–0.2 indicates a small effect; 0.2–0.6, a moderate effect; 0.6–0.8, a large effect; and > 0.8 a large to very large effect, depending on context. We used the Pustejovsky et al. (2024) online calculator with the significance level set to *p* < 0.05. As Pustejovsky et al. indicate that a minimum of three data points in each phase is needed to calculate Tau‐BC, effect size estimates are not provided for Wendy's follow‐up and baby probes on both her trained skills, and Sara's dressing follow‐up, and her cradle cap treatment follow‐up and baby probes. Sara's cradle cap baseline was the only one that showed a significant (decreasing) trend and was therefore automatically corrected in the calculator.

All reported Tau's were significant. Wendy's baseline‐training Tau‐BC's were 0.756, for giving a sponge bath and 0.943, for newborn ear and nose care. Sara's baseline‐training Tau‐BC's were 0.714 for giving a sponge bath; 0.745, for dressing; and 0.642, for treating cradle cap. Sara's baseline‐follow‐up with the infant simulator Tau‐BC was 0.714 for giving a sponge bath. Sara's baseline‐follow‐up Tau‐BC with the baby was 0.679, for sponge bath and 0.756 for dressing. In the context of the importance of increasing these critical newborn‐care skills, these effect sizes could be considered large changes (Vannest and Ninci 2015). Note that the Tau‐BC's for Sara's baby probes compared baseline probes with the infant simulator to follow‐up probes with the baby. Thus, these effect sizes may be considered to represent estimates of generalisation effects of Sara's performance from the infant simulator before training to the baby after training.

### 2.2 Parent Self‐Efficacy

Before training, the mean self‐efficacy rating score across the first 10 questions (see Table 3) was 3.85 (maximum score of 5), indicating that the participants were already fairly confident in their parenting abilities. After training, their mean score increased to 4.55, suggesting that the training was associated with a further increase in parent self‐efficacy. On Question 11—‘How confident are you in your abilities as a parent overall?’—Wendy's rating increased from 3 to 5, and Sara's rating went from 4 to 5.

### 2.3 Consumer Satisfaction

Both participants said they were highly satisfied (rating 5 out of 5) with the SBSPP training with the infant simulator. Wendy rated her satisfaction with the training of the two skills as 5. Sara rated training in giving a sponge bath and treating cradle cap as a 5, and dressing an infant training as a 3 (neither satisfied nor dissatisfied). The participants answered ‘yes’ to feeling comfortable asking the parent educators questions and that their questions were answered to their satisfaction. They also affirmed that the program was geared towards their learning style.

## 3 Discussion

This preliminary study showed that the Step‐by‐Step Parenting Program behavioural skills training using an infant simulator increased targeted newborn and infant care skills that transferred to the baby in expectant, first‐time parents with intellectual disabilities. As is common in pregnant women with intellectual disabilities, both participants experienced health complications to themselves or the fetus (see review by Feldman and Aunos 2020). Parenting self‐efficacy increased, and both parents were highly satisfied with the intervention.

The training results in this study of expectant mothers compare favourably to other SPSS studies in which parents received postnatal training with their babies (rather than an infant simulator). For instance, using the same multiple baseline designs as used in this study, Feldman et al. (1992a) taught 11 mothers with intellectual disabilities a total of 20 infant‐care skills like the skills taught in this study using similar checklists and training procedures. Mean scores increased from 58% in baseline to 90% during training to 91% in the 31‐week follow‐up. Note that the mothers in the current study started at a lower mean baseline (31%) than in the Feldman, Garrick et al. study, using an infant simulator rather than their familiar baby, and performed well on baby probes after training, despite having no prior experience caring for a newborn.

### 3.1 Parent Stress

As mothers with intellectual disability may report high stress (Feldman et al. 1997b), it is possible that the participants' stress related to becoming a new parent may have decreased during this training, as was found in a parenting intervention by Hodes et al. (2017). Although we did not directly measure the parents' stress, some of the comments on the consumer satisfaction forms suggest their stress was more manageable due to their participation in the prenatal training: ‘I'm a hands‐on learner. I like to be shown how to do something then I pick it up quick’; ‘I felt ready to call it quits due to my traumatic past…but I learned [through the program] to not give up throughout the process’; ‘I felt very motivated by my educator and husband due to positive reinforcement’; ‘I thoroughly enjoyed the process on how to become a mom’; ‘My favorite part was knowing what to do when my daughter comes’; ‘I felt prepared’; and ‘I did exactly what I learned with [the baby] and [it] helped a lot’.

### 3.2 Family Preservation

To the best of our knowledge, this is the first published study of the potential efficacy of parent training with expectant parents with intellectual disabilities using an infant simulator with transfer of skills to the newborn. This study contributes to the extant literature on interventions for parents with intellectual disabilities (Feldman and Aunos 2020). Parents with intellectual disabilities are more likely than virtually any other parents to have their newborn and infant children taken away (Keddell et al. 2023; McConnell et al. 2011, 2021). However, prenatal evidence‐based parenting skill training specifically designed for parents with cognitive disabilities (among other supports) may enhance important child‐care knowledge and skills that transfer to the baby and increase the chances of the parents keeping their babies. Indeed, as of this writing (over 13 months after the babies were born) both participating families have maintained custody of their children (they continued to receive SBSPP training on additional skills and other supports after they completed the study).

### 3.3 Infant Simulator

Using an infant simulator facilitates training before the baby is born. Even after birth, using the infant simulator during training may allow for more probes and training to be conducted than with the baby. For instance, while we may only ask the parent to practice bathing or diapering one time so as not to upset the infant, we can run multiple consecutive practices with the infant simulator. While we did use a programmable, life‐like infant simulator, we ran the study with the program off based on parent preference. Furthermore, the participants did not want to practice with the infant simulator between visits, and they told us that they did not practice the targeted skills with another baby replica or a baby. Yet, both participants improved their probe performance during training, for the most part, maintained skills ≥ 80% in follow‐up, and importantly, transferred their learned skills to their actual baby. Thus, a programmable infant simulator is probably unnecessary for the targeted skill training we implemented. Perhaps, a less expensive, nonprogrammable baby replica that closely resembles a real baby (e.g., has life‐like skin texture and heft) and could be left with the parents to practice at home (if they were willing) would be sufficient for this purpose.

### 3.4 Limitations

Limitations of this study include only two participants, and relatively few skills were trained because the participants were well into their pregnancy when recruited. Baseline Corrected Tau effect sizes should be interpreted cautiously given the relatively small number of data points (Pustejovsky et al. 2024; Tarlow 2017).

### 3.5 Future Research

More research is needed on prenatal training for parents with intellectual disabilities. Future studies could replicate this study with more participants and skills and test self‐directed learning. For instance, could a standardised set of task analysis videos slowly demonstrating how to perform a variety of child‐care skills result in skill improvements that transfer to the infant without additional BST components? Such findings would expand previous studies on self‐directed learning where parents with intellectual disabilities increased a variety of child‐care and safety skills by looking at step‐by‐step picture books and listening to audio recordings describing each step of several SBSPP checklists (Feldman 2004). Other studies could capitalise on the programmable potential of infant simulators to provide more realistic experiences and training to expectant parents with intellectual disabilities (if they were open to it), similar to what Höglund and Larsson (2019) did with special education high school students.

### 3.6 Practical Applications

This study has immediate practical applications for supporting expectant parents with intellectual disability. It provides an extension of the evidence‐based SBSPP collaborative parenting training model using an infant simulator to assess and teach newborn and infant skills that the parents may need to learn to adequately care for their newborn. As mentioned, it may not be necessary to use an expensive programmable infant simulator as both our participants learned skills with the simulator turned off. The encouraging results we obtained that the parents transferred the skills to their newborn suggest that parenting support provided prenatally may reduce the risk of the newborn being removed from the parents' care because of concerns that the parents lack the skills to care for the infant.

### 3.7 Conclusion

In conclusion, this study provided preliminary evidence that expectant, first‐time parents with intellectual disabilities can increase several newborn and infant skills with an evidence‐based training program specifically designed for parents with intellectual disabilities and transfer these skills to their baby. Of course, skill training on its own should be supplemented with additional services such as providing prenatal health information and monitoring in a manner that matches the parent's learning style. Comprehensive and accessible perinatal supports may reduce the risk of adverse parent and child peri‐ and postnatal outcomes, including child neglect and removal (Feldman and Aunos 2020).

## Author Contributions

Maurice Feldman: Made substantial contributions to conception and design, or acquisition of, or analysis and interpretation of data; was involved in drafting the manuscript or revising it critically for important intellectual content; gave final approval of the version to be published; participated sufficiently in the work to take public responsibility for appropriate portions of the content; and agrees to be accountable for all aspects of the work in ensuring that questions related to the accuracy or integrity of any part of the work are appropriately investigated and resolved. Amanda Cappon: Made substantial contributions to conception and design, or acquisition of, or analysis and interpretation of data; was involved in drafting the manuscript or revising it critically for important intellectual content; gave final approval of the version to be published; participated sufficiently in the work to take public responsibility for appropriate portions of the content; and agrees to be accountable for all aspects of the work in ensuring that questions related to the accuracy or integrity of any part of the work are appropriately investigated and resolved. Kay Corbier: Made substantial contributions to conception and design, or acquisition of, or analysis and interpretation of data; was involved in drafting the manuscript or revising it critically for important intellectual content; gave final approval of the version to be published; participated sufficiently in the work to take public responsibility for appropriate portions of the content; and agrees to be accountable for all aspects of the work in ensuring that questions related to the accuracy or integrity of any part of the work are appropriately investigated and resolved. Vicky Caruana: Made substantial contributions to conception and design, or acquisition of, or analysis and interpretation of data; was involved in drafting the manuscript or revising it critically for important intellectual content; gave final approval of the version to be published; participated sufficiently in the work to take public responsibility for appropriate portions of the content; and agrees to be accountable for all aspects of the work in ensuring that questions related to the accuracy or integrity of any part of the work are appropriately investigated and resolved. Mechane Laronde: Made substantial contributions to conception and design, or acquisition of, or analysis and interpretation of data; was involved in drafting the manuscript or revising it critically for important intellectual content; gave final approval of the version to be published; participated sufficiently in the work to take public responsibility for appropriate portions of the content; and agrees to be accountable for all aspects of the work in ensuring that questions related to the accuracy or integrity of any part of the work are appropriately investigated and resolved. Kendra Thomson: Made substantial contributions to conception and design, or acquisition of, or analysis and interpretation of data; was involved in drafting the manuscript or revising it critically for important intellectual content; gave final approval of the version to be published; participated sufficiently in the work to take public responsibility for appropriate portions of the content; and agrees to be accountable for all aspects of the work in ensuring that questions related to the accuracy or integrity of any part of the work are appropriately investigated and resolved.

## Ethics Statement

Ethics approval obtained from Research Ethics Boards of Durham College and Brock University. Four participants consented to participate in this study, understood, and signed the consent form, but only two participated in the study reported.

## Conflicts of Interest

Maurice Feldman receives compensation when he conducts training in the Step‐by‐Step Parenting Program but he received no compensation for the training conducted for this study. Vicky Caruna and Mechane Laronde received compensation as the parent educators in this study. The other authors report no conflicts of interest.

## Data Availability

Data are unavailable due to participant privacy protection.
